# Prevalence and determinants of high-risk obstructive sleep apnea among hypertensive patients in referral hospitals of the Amhara Region, Northwest Ethiopia, 2022

**DOI:** 10.3389/frsle.2025.1554653

**Published:** 2025-05-16

**Authors:** Meseret Kassaw, Bezawit Mulat Ayal, Dagimawi Chilot, Kassa Abebaw, Baye Ashenef

**Affiliations:** ^1^Department of Biomedical Sciences, School of Medicine, Debre Markos University, Debre Markos, Ethiopia; ^2^Department of Human Physiology, College of Medicine and Health Sciences, University of Gondar, Gondar, Ethiopia; ^3^Department of Biomedical Sciences, School of Medicine, Injbara University, Injbara, Ethiopia

**Keywords:** hypertension, obstructive sleep apnea, associated factors, Ethiopia, breathing

## Abstract

**Background:**

Obstructive sleep apnea results from intermittent airway collapse during sleep. Despite its health risks, the prevalence and associated factors of OSA among hypertensive patients in Ethiopia remain unexplored.

**Objective:**

This study assessed the prevalence and associated factors of high-risk OSA among hypertensive patients in referral hospitals within the Amhara Region, Northwest Ethiopia, in 2022.

**Methods:**

A cross-sectional study was conducted in selected referral hospitals from 21 April to 14 June 2022. A systematic random sampling technique was employed. Data were collected through structured, pretested, interviewer-administered questionnaires, file reviews, and physical examinations. Data were entered in Epi-Data 4.6 and analyzed using Stata 14. Logistic regression was performed, and variables with *p* < 0.05 were regarded as significantly associated with high-risk OSA.

**Results:**

Of the 412 participants (97% response rate), the mean age was 58.95 ± 12.6 years, with 55.1% being female. The prevalence of high-risk OSA was determined to be 43.93% (95% CI: 39.2–48.8). Significant factors included age > 65 years (AOR = 8.00, 95% CI: 4.48–14.14), diabetes mellitus (AOR = 4.7, 95% CI: 2.48–8.94), male sex (AOR = 4.2, 95% CI: 2.38–7.45), and large neck circumference (AOR = 3.13, 95% CI: 1.27–7.74).

**Conclusion:**

High-risk OSA is prevalent among hypertensive patients, particularly in older males and those with diabetes or a large neck circumference. Routine OSA screening should be integrated into hypertension care. Future studies should utilize gold-standard tools and explore cause-and-effect relationships.

## Introduction

Obstructive sleep apnea (OSA) is a sleep disorder characterized by repeated upper airway obstruction during sleep due to intermittent airway collapse (Bacci et al., 1992). This condition arises from factors that reduce the pharyngeal space, leading to disrupted airflow (Dempsey et al., 2010). Common symptoms include snoring, daytime sleepiness, restles sleep, morning fatigue, and headaches (Phillips and O'Driscoll, 2013). The gold standard for diagnosis is polysomnography; however, in its absence, standardized assessment tools such as the Berlin and STOP-BANG Questionnaires are effective for risk assessment (Tintinger et al., 2011; Chung et al., 2008, 2016). OSA severity is classified by the apnea-hypopnea index (AHI) as mild (5–15 events/h), moderate (16–30 events/h), or severe (>30 events/h) (Prejbisz et al., 2014).

OSA is a significant global health concern, affecting approximately one billion individuals (Prejbisz et al., 2014). It impacts 4–9% of middle-aged men and 1–2% of middle-aged women, with prevalence reaching 20–30% in obese individuals (Phillips and O'Driscoll, 2013). Among adults aged 30–69 years, an estimated 936 million have mild to severe OSA, while 425 million have moderate to severe OSA (Hedner et al., 2006). Epidemiological studies reveal varying prevalence rates: 73% in Sweden (Hedner et al., 2006), 58.1% in Brazil (Bacci et al., 1992), 24% in India (Kareem et al., 2020), 70.5% in China (Cai et al., 2017), 32.38% in Thailand (Jinchai et al., 2020), 42.1% in Tanzania (Pallangyo et al., 2021), and 52% in hypertensive individuals in Nigeria (Akintunde et al., 2012).

Key risk factors for OSA include obesity, chronic illnesses, smoking, and alcohol consumption (Tintinger et al., 2011; Lin et al., 2012; Kasai et al., 2012; Aurora and Punjabi, 2013; Abuyassin et al., 2015). Severe OSA (AHI >30) significantly elevates the risk of cardiovascular events such as myocardial infarction, stroke, hypertension, and arrhythmias (Korostovtseva et al., 2011; Kario, 2009; Shah et al., 2010). Chronic hypoxia in OSA leads to renal damage, increasing the risk of chronic kidney disease (Fu et al., 2016). Additionally, OSA contributes to metabolic dysregulation, oxidative stress, heightened sympathetic activity, lipolysis, inflammation, and insulin resistance, exacerbating type 2 diabetes and obesity (Gaines et al., 2018). It is also linked to secondary and resistant hypertension (Kario, 2009). Overall, OSA is associated with reduced quality of life, substantial morbidity, and increased cardiovascular mortality risk, with affected individuals being 4–5 times more likely to experience fatal cardiovascular events (Tintinger et al., 2011; Young et al., 2008). Additionally, mortality rates are higher in the severe OSA group (41%) compared to the mild and moderate OSA group (29%) (Won et al., 2013). Continuous positive airway pressure (CPAP) remains the primary treatment, significantly reducing OSA-related complications (Litvin et al., 2013).

Although OSA is known to reduce the quality of life and increase morbidity and mortality, research on OSA in Africa is limited, and there are no documented studies in Ethiopia examining its prevalence and associated factors among hypertensive patients. This study seeks to fill this gap by providing valuable baseline data for future research and offering a foundation for policymakers to address this critical health challenge.

## Methods and materials

### Study setting and period

The study was conducted from April 21 to June 14, 2022, at Felege Hiwot, Debre Markos, and Debre Tabor referral hospitals in the Amhara Region, Ethiopia. These hospitals serve over 25 million people, including about 2,500 hypertensive patients receiving follow-up care (unpublished data).

### Study design and period

An institutional-based cross-sectional study was conducted among hypertensive patients in the Amhara region in selected referral hospitals in Ethiopia. Of 423 invited patients, 412 completed the questionnaire, but 11 were excluded due to incomplete responses. The source population included all hypertensive patients in follow-up care (every 3 months) at the selected hospitals, while the study population comprised those attending Felege Hiwot, Debre Markos, and Debre Tabor referral hospitals during data collection.

### Inclusion and exclusion criteria

Hypertensive patients (BP >140/90 mmHg), aged ≥18 years, who gave voluntary consent were included. However, critically ill patients or those with COPD or psychiatric disorders were excluded.

### Sample size determination

The sample size was calculated using a single population proportion formula.


$$\begin{array}{l} \;n=\frac{{\left(Z_{\alpha /2}\right)}^{2}\times \;p\left(1-p\right)}{\;d^{2}}=\frac{{\left(1.96\right)}^{2}\times \;\left(0.5\right)\left(0.5\right)}{{\left(0.05\right)}^{2}}\\ \;=384\;+10\%=423 \end{array}$$

#### Assumption

*n* = sample size, *P* = proportion of the risk of OSA = 50% since the study on the risk of OSA among visually hypertensive patients was not conducted in the study area, *d* = Margin of sampling error tolerated-5% (0.05), α = Critical value at 95% confidence interval of certainty (1.96).

### Sampling procedure

A combination of simple and systematic random sampling was used. Three hospitals (Felege Hiwot, Debre Markos, and Debretabor Referral Hospitals) were randomly selected by the lottery method. A sampling frame of hypertensive patients was created from chronic disease clinic records. Systematic random sampling was applied, selecting every 2nd patient (*K* = 6, derived from 2,500 hypertensive patients divided by a sample size of 423). The starting point (patient 2) was chosen by lottery, and participants were proportionally allocated across hospitals for representativeness.

### Study variables

#### Dependent variable

High risk of obstructive sleep apnea (yes/no).

#### Independent variables

##### Socio-demographic factors

Age, sex, residence, religion, education, occupation.

##### Behavioral factors

Smoking, Alcohol consumption.

##### Clinical factors

Heart failure, Kidney failure, Type 2 Diabetes Mellitus.

##### Anthropometric measurements

Body Mass Index (BMI), Neck circumference, Waist circumference, Physical inactivity, Family history of snoring.

### Data collection procedure and data collection tools

Data were collected using the STOP-BANG questionnaire, an eight-item tool assessing OSA risk based on snoring, daytime sleepiness, observed apnea, hypertension, BMI, age (≥50), neck circumference, and male gender (Chiu et al., 2017; Abumuamar et al., 2018). It is administered by an interviewer, takes 2–5 min, and has over 80% sensitivity but <50% specificity, effectively identifying high-risk individuals (Abumuamar et al., 2018; Luo et al., 2014). A STOP-BANG score of 0 to 2 indicates a low risk of obstructive sleep apnea, a score of 3 to 4 suggests an intermediate risk, and a score of 5 to 8 reflects a high risk of the condition (Yang and Chung, 2013 #186). Additionally, data were gathered through medical chart reviews and physical examinations, which included measurements of weight, height, neck circumference, and waist circumference. Weight was assessed using a digital scale (±0.5 kg) while participants wore light clothing and no shoes, and height was measured with a stadiometer, ensuring participants stood erect, barefoot, with their heels against a wall or board (Douketis et al., 2005).

BMI was calculated as weight divided by height squared (kg/m^2^). Neck circumference was measured with non-elastic tape at the cricothyroid level, and waist circumference at the midpoint between the lower costal margin and the iliac crest was recorded to the nearest 0.5 cm (Douketis et al., 2005). Clinical factors were obtained from the patient file.

### Data processing and analysis

After data collection, the dataset was cleaned, edited, and entered into Epi Data 4.6, then exported to Stata 14.0 for analysis. Descriptive statistics summarized the study population. Bi-variable and multivariable logistic regressions examined associations, with variables (*p* < 0.25) from the bivariable analysis included in the multivariable model. Statistical significance was set at *p* < 0.05. Multicollinearity among selected independent variables was checked, and the variance inflation 1.58 factor was found to be acceptable (<2), and the Hosmer-Lemeshow test (χ^2^ = 17.95, *p* = 0.16) confirmed a good model fit. A *post-hoc* power analysis was conducted to assess the study's statistical power to identify significant associations between high-risk OSA and its determinants among hypertensive patients, and with a final sample size of 412, an observed odds ratio of 1.5, and a significance level of 0.05, the calculated power was 0.847. The sensitivity analysis was conducted using Cook's distance and leverage, which would be below the typical threshold of 1.

### Data quality assurance

A 1-day training session was held for data collectors and supervisors by the principal investigator, covering data collection methods, patient interaction, and maintaining privacy and confidentiality. Two trained diploma nurses collected data from the target population, with daily reviews by the supervisor and principal investigator to ensure consistency and completeness. A pretest was conducted at Tibebe Geon Specialized Hospital with 30 participants (5% of the total sample size) before the main data collection. During the interview, all participants showed a clear understanding of the questionnaire.

## Results

### Socio-demographic characteristics of study participants

A total of 412 hypertensive patients participated, with a response rate of 97%. The mean age was 58.95 ± 12.6 years. Of the participants, 227 (55.1%) were female, and 260 (63.1%) lived in urban areas. Most identified as Orthodox Christians and were married. Additionally, 144 (37.95%) were illiterate, and 163 (39.56%) were farmers (Table 1).

**Table 1:** Socio-demographic characteristics of hypertensive patients in selected referral hospitals, Amhara region, Ethiopia, 2022(*n* = 412).

**Variable**	**Category**	**Frequency**	**Percentage (%)**
Sex	Male	185	44.9
	Female	227	55.1
Age (years)	≤ 65	257	62.38
	>65	155	37.62
Residence	Urban	260	63.11
	Rural	152	36.89
Religion	Orthodox	327	79.37
	Muslim	58	14.08
	Protestant and Catholic	27	6.55
Educational status	Unable to read and write	144	34.95
	Able to read and write	113	27.43
	Primary	55	13.35
	Secondary	49	11.89
	Diploma and above	51	12.38
Occupation	Farmer	164	39.81
	Government employee	35	8.5
	Merchant	70	16.99
	Housewife	102	24.76
	Retire	41	9.95
Marital status	Unmarried	8	1.94
	Married	335	81.31
	Divorced	23	5.58
	Widowed	46	11.11

### Clinical and behavioral characteristics of study participants

Among hypertensive patients, 102 (24.76%) were also diabetic. The majority, 408 (99%), were non-smokers, and 130 (28.1%) had a history of alcohol consumption. Most participants, 310 (75.24%), were physically inactive. Only 54 (13.11%) had a family history of snoring. The average weight was 67.1 ± 11.7 kg, height 1.61 ± 0.07 m, neck circumference 35.6 ± 3.9 cm, and waist circumference 91.6 ± 10.9 cm. The average BMI was 26 ± 4.25, while mean systolic and diastolic blood pressures were 132 ± 26.1 mmHg and 80.37 ± 9.56 mmHg, respectively (Table 2).

**Table 2:** Clinical and behavioral characteristics of hypertensive patients in selected referral hospitals, Amhara region, Ethiopia, 2022 (*n* = 412).

**Variable**	**Frequency**	**Percentage (%)**
**Comorbidities**		
Diabetes mellitus	102	24.76
Kidney failure	64	15.53
Heart failure	62	15.05
**Cigarette smoker**		
No	408	99
Yes	4	1
**Alcohol drinking**		
No	239	59
Current alcohol drinkers	43	12.9
Former alcohol drinker	130	28.1
**BMI**		
<18.5 kg/m^2^	16	3.88
18.5–24.99 kg/m^2^	144	34.95
25–29.99 kg/m^2^	186	45.15
>30 kg/m^2^	66	16.02

### Findings of *post-hoc* power analysis

The *post-hoc* power analysis demonstrated that the power to detect significant associations between key determinants and high-risk OSA was 0.85, surpassing the typical 0.80 threshold for adequate power and confirming that the sample size provided enough power for prevalence estimation, ensuring the statistical robustness of the observed high-risk OSA prevalence rates.

### Prevalence of risk of obstructive sleep apnea

Among hypertensive participants, 43.93% (95% CI: 39.52–48.8) were at high risk of OSA, while 56.07% (95% CI: 51.2–60.8) were at low risk.

### Subgroup analysis results: high vs. low risk of OSA

The risk of obstructive sleep apnea (OSA) was significantly influenced by various demographic and clinical factors: males had a higher risk than females (61.1 vs. 29.95%), with low risk more prevalent in females (70.05%) than males (38.9%); individuals over 65 years exhibited a higher risk (74.84%) compared to those 65 years or younger (25.3%), who had a greater proportion in the low-risk category (74.7 vs. 25.16%); urban residents had a slightly higher high-risk rate (47.3%) and slightly lower low-risk rate (52.7%) than rural residents (38.16 and 61.84%, respectively). Diabetic patients were more likely to be at high risk (74.5%) than non-diabetics (33.9%), with non-diabetics more commonly in the low-risk group (66.1 vs. 25.5%); respondents with renal failure (64%) or heart failure (58%) had a higher risk than those without these conditions (40.23 and 42.4%, respectively), while the latter had greater low-risk proportions (59.77 and 58.6%, respectively); alcohol drinkers showed a higher risk (47.95%) than non-drinkers (39.38%), who had a greater proportion of low-risk individuals (60.62 vs. 52.05%); a family history of snoring was associated with a much higher risk of OSA (85.98%) than no family history (16.13%); individuals with a BMI ≥30 kg/m^2^ had a high risk (65.15%), whereas those with BMI 18.5–24.9 kg/m^2^ (76.39%) and <18.5 kg/m^2^ (68.75%) had low-risk; those with neck circumference >40 cm had a high risk (77.8%) compared to those with ≤ 40 cm (61.18%) who were had low-risk; participants with waist circumference >102 cm had a higher risk (69.74%) than those with ≤ 102 cm, who more frequently had a low risk (61.9 vs. 30.26%); and individuals with systolic blood pressure (SBP) >140 mmHg had a higher OSA risk (55%), while those with SBP <140 mmHg had low risk (58.74%; Table 3).

**Table 3:** Bivariable and multivariable binary logistic regression analysis of factors associated with high risk of OSA among hypertensive patients in selected referral hospitals, Amhara region, Ethiopia, 2022(*n* = 412).

**Variables**		**High-risk OSA**		**COR (95% CI)**	***P*-value**	**AOR (95% CI)**	***P*-value**
		**Yes (%)**	**No (%)**				
Sex	Female	68 (29.95)	159 (70.05)	1	1	1	
	Male	113 (61.1)	72 (38.9)	3.7 (2.43–5.52)	0.08	4.2 (2.38–7.45)	0.001
Age	≤ 65 years	65 (25.3)	192 (74.7)	1	1	1	
	>65 years	116(74.84)	39 (25.16)	8.8 (5.55–13.9)	0.03	8 (4.48–14.14)	0.01
Residence	Rural	58 (38.16)	94 (61.84)	1	1	1	
	Urban	123 (47.3)	137 (52.7)	1.45 (0.97–2.19)	0.049	0.9 (0.53–2.00)	0.527
DM	No	105 (33.9)	205 (66.1)	1	1	1	
	Yes	76 (74.5)	26 (25.5)	5.7 (3.45–9.44)	0.038	4.7 (2.48–8.94)	0.003
Kidney failure	No	140 (40.23)	208 (59.77)	1	1		
	Yes	41 (64)	23 (36)	2.65 (1.5–4.6)	0.158		0.247
Heart failure	No	145 (42.4)	205 (58.6)	1	1	1	
	Yes	36 (58)	26 (42)	1.96 (1.13–3.38)	0.223	1.13 (0.52–2.45)	0.382
Alcohol drinking	No	76 (39.38)	117 (60.62)	1	1	1	
	Yes	105 (47.95)	114 (52.05)	1.35 (0.9, 2.0)	0.07	1.06 (0.6, 1.84)	0.200
Family history snoring	No	141 (85.98)	23 (14.02)	1	1	1	
	Yes	40 (16.13)	208 (83.87)	2.57 (1.47, 4.47)	0.14	1.12 (0.52, 2.4)	0.052
BMI	<18.5 Kg/m^2^	5 (31.25)	11 (68.75)	1	1	1	
	18.5–24.9 Kg/m^2^	34 (23.61)	110 (76.39)	0.68 (0.22, 2.1)	0.12	0.84 (0.89, 3.7)	0.829
	24.99–29.9 Kg/m^2^	99 (53.22)	87 (46.78)	2.5 (0.84, 7.45)	0.25	2.34 (0.53, 10.3)	0.810
	≥30 Kg/m^2^	43 (65.15)	23 (34.85)	4.11 (1.27, 14.3)	0.16	3.65 (0.7, 19.07)	0.186
Neck circumference	≤ 40 cm	139 (38.82)	219 (61.18)	1	1	1	
	>40 cm	42 (77.8)	12 (22.2)	5.5 (2.8, 10.84)	0.001	3.13 (1.27, 7.7)	0.005
Waist circumference	≤ 102 cm	128 (38.1)	208 (61.9)	1		1	
	>102 cm	53 (69.74)	23 (30.26)	3.74 (2.19, 6.4)	0.21	1.32 (0.59, 2.97)	0.176
SBP	<140 mmHg	137 (41.26)	195 (58.74)	1	1	1	
	>140 mmHg	44 (55)	36 (45)	1.73 (1.06, 2.84)	0.057	0.93 (0.46, 1.84)	0.159

COR, Crude odds ratio; AOR, Adjusted odds ratio; CI, Confidence interval.

### Factors associated with the risk of OSA

In the bivariable binary logistic regression analysis, factors such as age, sex, residence, diabetes mellitus, heart failure, kidney failure, systolic blood pressure, family history of snoring, alcohol consumption, BMI, neck circumference, and waist circumference were associated with an increased risk of obstructive sleep apnea (OSA). However, in the multivariable analysis, significant associations with a high risk of OSA were being over 65 years old, male, having diabetes mellitus, and having a large neck circumference.

Those over 65 had an 8fold higher risk of OSA compared to those under 65 (AOR = 8.00, 95% CI: 4.48–14.14). Individuals with diabetes were 4.7 times more likely to be at high risk for OSA (AOR = 4.7, 95% CI: 2.48–8.94). Men had a 4.2-fold higher likelihood of OSA risk than women (AOR = 4.2, 95% CI: 2.38–7.45). Additionally, those with a neck circumference over 40 cm had 3.13 times higher odds of OSA risk (AOR = 3.13, 95% CI: 1.27–7.74; Table 3).

In our data, the sensitivity analysis revealed that the maximum values of Cook's distance and leverage were found to be extremely less than one (0.03–0.2).

## Discussion

This study assessed the risk of obstructive sleep apnea (OSA) and associated factors among hypertensive patients in referral hospitals within the Amhara Regional State.

The prevalence of high-risk OSA in this study was 43.93% (95% CI: 39.2–48.8), which was higher than the study done in Thailand at 32.38% (Jinchai et al., 2020) and India at 23.8% (Kareem et al., 2020). This discrepancy may be due to the larger sample size in this study compared to the Thailand and India study, which focused on younger hypertensive patients under 35 years with 118 participants. The higher illiteracy rate and limited education and healthcare access among participants likely contributed to an overestimation of high-risk obstructive sleep apnea prevalence. This study, with a larger sample and primarily older participants, may account for the difference.

Conversely, the prevalence observed in this study was lower than in studies conducted in Nigeria (52%) (Akintunde et al., 2012), Brazil (58.1%) (Bacci et al., 1992), Sweden (73%) (Hedner et al., 2006), and China (70.5% ) (Cai et al., 2017). These differences may stem from variations in assessment tools. This study used the STOP-BANG questionnaire, a practical screening tool, while studies in Sweden and China used polysomnography, the gold standard for OSA diagnosis, and those in Nigeria and Brazil used the Berlin questionnaire. The study populations may also have contributed to these variations; for example, the Chinese study had a larger sample with mostly male participants, while this study had a smaller sample with more female participants.

This finding aligns closely with a study conducted in Tanzania, which reported a prevalence of 42.1% (Pallangyo et al., 2021).

Age was found to be a significant factor, with older individuals having eight times higher odds of being at high risk, as supported by studies in Nigeria (Akintunde et al., 2012) and Brazil (Bacci et al., 1992). This association may be due to anatomical changes in the upper airway, increased pharyngeal resistance, airway oscillations, and sleep instability with aging (Levy et al., 1996).

The study found that males were 4.2 times more likely to have a high risk of OSA than females, aligning with studies from Sweden (Bacci et al., 1992) and China (Cai et al., 2017). This difference may be due to sex hormones like progesterone, which helps maintain airway patency in females by enhancing airway dilator muscle activity during sleep (Sigurð*ardóttir et al., 2022). Additionally, males tend to have more fat around the neck, increasing airway load and promoting pharyngeal collapse during sleep (Whittle et al., 1999).

Comorbid diabetes mellitus (DM) was linked to a 4.7-fold higher risk of high-risk OSA, as supported by studies in Nigeria (Akintunde et al., 2012) and Tanzania (Pallangyo et al., 2021). This association may be due to DM-induced peripheral autonomic neuropathy, which impairs upper airway muscle function, mechanoreceptor activation, and ventilatory control, leading to reduced airway diameter and increased airway obstruction risk (Vinik et al., 2003).

Finally, a large neck circumference was strongly linked to high-risk OSA, as supported by studies in Nigeria (Akintunde et al., 2012), Tanzania (Pallangyo et al., 2021), and India (Kareem et al., 2020). This association is likely due to subcutaneous fat in the neck, which increases airway pressure and promotes pharyngeal collapse during sleep.

This study relied on participants' recall for many questions, which may introduce recall bias. As a cross-sectional study, it cannot establish cause-and-effect relationships. Moreover, the absence of a confirmatory polysomnography test may have led to an inaccurate estimation of OSA risk.

## Conclusion and recommendations

Nearly half of the hypertensive patients in this study were at high risk for obstructive sleep apnea (OSA), with significant associations observed for male gender, diabetes mellitus, age over 65, and neck circumference above 40 cm. These findings highlight the need for routine OSA screening in referral hospitals, particularly for hypertensive patients presenting with symptoms like daytime sleepiness, fatigue, or snoring. Future research should employ gold-standard screening methods and stronger study designs to clarify causal links while also considering factors such as medication use, physical activity, diet, sleep duration, and daytime sleepiness. We further recommend using validated tools, random sampling when feasible, and including control groups from non-conflict areas to enhance data quality and generalizability.

## Data Availability

The datasets presented in this study can be found in online repositories. The names of the repository/repositories and accession number(s) can be found in the article/supplementary material.

## References

[B1] Abumuamar A. M. Dorian P. Newman D. Shapiro C. M. (2018). The STOP-BANG questionnaire shows an insufficient specificity for detecting obstructive sleep apnea in patients with atrial fibrillation. J. Sleep Res. 27:e12702. 10.1111/jsr.12702 29682848

[B2] Abuyassin B. Sharma K. Ayas N. T. Laher I. (2015). Obstructive sleep apnea and kidney disease: a potential bidirectional relationship? J. Clin. Sleep Med. 11, 915–924. 10.5664/jcsm.4946 25845900 PMC4513269

[B3] Akintunde A. A. Okunola O. O. Oluyombo R. Oladosu Y. O. Opadijo O. G. OSnoring obstructive sleep apnoea syndrome among hypertensive Nigerians: prevalence clinical correlates. Pan Afr. Med. J. (2012). 11, 1071–1078. 22655109 PMC3361213

[B4] Aurora R. N. Punjabi N. M. (2013). Obstructive sleep apnoea and type 2 diabetes mellitus: a bidirectional association. Lancet Respir. Med. 1, 329–338. 10.1016/S2213-2600(13)70039-0 24429158

[B5] Bacci M. R. Emboz J. N. M. Alves B. Veiga G. L. D. Murad N. Meneghini A.. (1992). Obstructive sleep apnea syndrome and sleep quality in hypertensive patients. Rev. Assoc. Med. Bras. 63, 1055–1060. 10.1590/1806-9282.63.12.1055 29489985

[B6] Cai A. Zhou Y. Zhang J. Zhong Q. Wang R. Wang L.. (2017). Epidemiological characteristics and gender-specific differences of obstructive sleep apnea in a Chinese hypertensive population: a cross-sectional study. BMC Cardiovasc. Disord. 17, 1–7. 10.1186/s12872-016-0447-4 28056799 PMC5217623

[B7] Chiu H. Y. Chen P. Y. Chuang L. P. Chen N. H. Tu Y. K. Hsieh Y. J.. (2017). Diagnostic accuracy of the Berlin questionnaire, STOP-BANG, STOP, and Epworth sleepiness scale in detecting obstructive sleep apnea: a bivariate meta-analysis. Sleep Med. Rev. 36, 57–70. 10.1016/j.smrv.2016.10.004 27919588

[B8] Chung F. Abdullah H. R. Liao P. (2016). STOP-bang questionnaire: a practical approach to screen for obstructive sleep apnea. Chest 149, 631–638. 10.1378/chest.15-0903 26378880

[B9] Chung F. Yegneswaran B. Liao P. Chung S. A. Vairavanathan S. Islam S.. (2008). STOP questionnaire: a tool to screen patients for obstructive sleep apnea. Anesthesiology 108, 812–821. 10.1097/ALN.0b013e31816d83e4 18431116

[B10] Dempsey J. A. Veasey S. C. Morgan B. J. O'Donnell C. P. (2010). Pathophysiology of sleep apnea. Physiol. Rev. 90, 47–112. 10.1152/physrev.00043.2008 20086074 PMC3970937

[B11] Douketis J. D. Paradis G. Keller H. Martineau C. (2005). Canadian guidelines for body weight classification in adults: application in clinical practice to screen for overweight and obesity and to assess disease risk. CMAJ 172, 995–998. 10.1503/cmaj.045170 15824401 PMC556034

[B12] Fu Q. Colgan S. P. Shelley C. S. (2016). Hypoxia: the force that drives chronic kidney disease. Clin. Med. Res. 14, 15–39. 10.3121/cmr.2015.1282 26847481 PMC4851450

[B13] Gaines J. Vgontzas A. N. Fernandez-Mendoza J. Bixler E. O. (2018). Obstructive sleep apnea and the metabolic syndrome: the road to clinically meaningful phenotyping, improved prognosis, and personalized treatment. Sleep Med. Rev. 42, 211–219. 10.1016/j.smrv.2018.08.009 30279095 PMC6221996

[B14] Hedner J. Bengtsson-Boström K. Peker Y. Grote L. Råstam L. Lindblad U.. (2006). Hypertension prevalence in obstructive sleep apnoea and sex: a population-based case-control study. Eur. Respir. J. 27, 564–570. 10.1183/09031936.06.00042105 16507857

[B15] Jinchai J. Khamsai S. Chattakul P. Limpawattana P. Chindaprasirt J. Chotmongkol V.. (2020). How common is obstructive sleep apnea in young hypertensive patients? Intern. Emerg. Med. 15, 1005–1010. 10.1007/s11739-019-02273-3 31970622

[B16] Kareem O. Tanvir M. Bader G. N. (2020). Prevalence of high-risk obstructive sleep apnoea by Berlin questionnaire in patients with hypertension: a study from a tertiary care hospital. Sleep Sci. Pract. 4, 1–9. 10.1186/s41606-020-00052-032395635

[B17] Kario K. (2009). Obstructive sleep apnea syndrome and hypertension: ambulatory blood pressure. Hypertens. Res. 32, 428–432. 10.1038/hr.2009.56 19494815

[B18] Kasai T. Floras J. S. Bradley T. D. (2012). Sleep apnea and cardiovascular disease: a bidirectional relationship. Circulation 126, 1495–1510. 10.1161/CIRCULATIONAHA.111.070813 22988046

[B19] Korostovtseva L. S. Sviryaev Y. V. Zvartau N. E. Konradi A. O. Kalinkin A. L. (2011). Prognosis and cardiovascular morbidity and mortality in a prospective study of hypertensive patients with obstructive sleep apnea syndrome in St Petersburg, Russia. Med. Sci. Monit. Int. Med. J. Exp. Clin. Res. 17, Cr146–53. 10.12659/MSM.881448 PMC3524738 21358601

[B20] Levy P. Pepin J. Malauzat D. Emeriau J. Leger J. (1996). Is sleep apnea syndrome in the elderly a specific entity? Sleep 19, S29–38. 10.1093/sleep/19.suppl_3.S29 8723373

[B21] Lin Y. N. Li Q. Y Zhang X. J. (2012). Interaction between smoking and obstructive sleep apnea: not just participants. Chin. Med. J. 125, 3150–3156. 22932197

[B22] Litvin A. Y. Sukmarova Z. N. Elfimova E. M. Aksenova A. V. Galitsin P. V. Rogoza A. N.. (2013). Effects of CPAP on “vascular” risk factors in patients with obstructive sleep apnea and arterial hypertension. Vasc. Health Risk Manag. 9, 229–235. 10.2147/VHRM.S40231 23690688 PMC3656895

[B23] Luo J. Huang R. Zhong X. Xiao Y. Zhou J. (2014). STOP-bang questionnaire is superior to Epworth sleepiness scales, Berlin questionnaire, and STOP questionnaire in screening obstructive sleep apnea-hypopnea syndrome patients. Chin. Med. J. 127, 3065–3070. 10.3760/cma.j.issn.0366-6999.20133003 25189946

[B24] Pallangyo P. Mgopa L. R. Mkojera Z. Komba M. Millinga J. Misidai N.. (2021). Obstructive sleep apnea and associated factors among hypertensive patients attending a tertiary cardiac centre in Tanzania: a comparative cross-sectional study. Sleep Sci. Pract. 5, 1–11. 10.1186/s41606-021-00069-z

[B25] Phillips C. L. O'Driscoll D. M. (2013). Hypertension, and obstructive sleep apnea. Nat. Sci. Sleep 5, 43–52. 10.2147/NSS.S34841 23750107 PMC3666153

[B26] Prejbisz A. Florczak E. Pregowska-Chwała B. Klisiewicz A. Kuśmierczyk-Droszcz B. Zieliński T.. (2014). Relationship between obstructive sleep apnea and markers of cardiovascular alterations in never-treated hypertensive patients. Hypertens. Res. 37, 573–579. 10.1038/hr.2014.43 24621467

[B27] Shah N. A. Yaggi H. K. Concato J. Mohsenin V. (2010). Obstructive sleep apnea as a risk factor for coronary events or cardiovascular death. Sleep Breath 14, 131–136. 10.1007/s11325-009-0298-7 19777281

[B28] Sigurð*ardóttir E. S. Gislason T. Benediktsdottir B. Hustad S. Dadvand P. Demoly P.. (2022). Female sex hormones and symptoms of obstructive sleep apnea in European women of a population-based cohort. PLoS ONE 17:e0269569. 10.1371/journal.pone.0269569 35731786 PMC9216532

[B29] Tintinger G. R. Pretorius L. Labadarios D. (2011). Obstructive sleep apnoea and obesity. South Afr. J. Clin. Nutr. 24, 174–177. 10.1080/16070658.2011.11734384

[B30] Vinik A. I. Maser R. E. Mitchell B. D. Freeman R. (2003). Diabetic autonomic neuropathy. Diabetes Care 26, 1553–1579. 10.2337/diacare.26.5.1553 12716821

[B31] Whittle A. T. Marshall I. Mortimore I. L. Wraith P. K. Sellar R. J. Douglas N. J.. (1999). Neck soft tissue and fat distribution: comparison between normal men and women by magnetic resonance imaging. Thorax 54, 323–328. 10.1136/thx.54.4.323 10092693 PMC1745454

[B32] Won C. H. Chun H. J. Chandra S. M. Sarinas P. S. Chitkara R. K. Heidenreich P. A.. (2013). Severe obstructive sleep apnea increases mortality in patients with ischemic heart disease and myocardial injury. Sleep Breath 17, 85–91. 10.1007/s11325-012-0653-y 22294346

[B33] Yang Y. Chung F. (2013). A screening tool of obstructive sleep apnea: STOP-bang questionnaire. Sleep Med. Clin. 8, 65–72.

[B34] Young T. Finn L. Peppard P. E. Szklo-Coxe M. Austin D. Nieto F. J.. (2008). Sleep-disordered breathing and mortality: eighteen-year follow-up of the Wisconsin sleep cohort. Sleep 31, 1071–1078. 18714778 PMC2542952

